# GPER deletion triggers inhibitory effects in triple negative breast cancer (TNBC) cells through the JNK/c-Jun/p53/Noxa transduction pathway

**DOI:** 10.1038/s41420-023-01654-0

**Published:** 2023-09-26

**Authors:** Francesca Cirillo, Marianna Talia, Maria Francesca Santolla, Michele Pellegrino, Domenica Scordamaglia, Asia Spinelli, Salvatore De Rosis, Francesca Giordano, Lucia Muglia, Azzurra Zicarelli, Marika Di Dio, Damiano Cosimo Rigiracciolo, Anna Maria Miglietta, Gianfranco Filippelli, Ernestina Marianna De Francesco, Antonino Belfiore, Rosamaria Lappano, Marcello Maggiolini

**Affiliations:** 1 Department of Pharmacy, Health and Nutritional Sciences, University of Calabria, 87036 Rende, Italy; 2 Department of Experimental Oncology, IEO, European Institute of Oncology IRCCS, Via Adamello 16, 20139 Milano, Italy; 3 Breast and General Surgery Unit, Regional Hospital Cosenza, 87100 Cosenza, Italy; 4 Oncology Department, Hospital of Paola, 87100 Cosenza, Italy; 5 Endocrinology, Department of Clinical and Experimental Medicine, University of Catania, Garibaldi-Nesima Hospital, 95122 Catania, Italy

**Keywords:** Breast cancer, Endocrine cancer

## Abstract

The G protein-coupled estrogen receptor (GPER) mediates estrogen action in different pathophysiological conditions, including cancer. GPER expression and signaling have been found to join in the progression of triple-negative breast cancer (TNBC), even though controversial data have been reported. In present study, we aimed at providing new mechanistic and biological discoveries knocking out (KO) GPER expression by CRISPR/Cas9 technology in MDA-MB-231 TNBC cells. GPER KO whole transcriptome respect to wild type (WT) MDA-MB-231 cells was determined through total RNA sequencing (RNA-Seq) and gene ontology (GO) enrichment analysis. We ascertained that anti-proliferative and pro-apoptotic gene signatures characterize GPER KO MDA-MB-231 cells. Thereafter, we determined that these cells exhibit a reduced proliferative, clonogenic and self-renewal potential along with an increased mitochondria-dependent apoptosis phenotype. In addition, we recognized that decreased cAMP levels trigger the JNK/c-Jun/p53/Noxa axis, which in turn orchestrates the pro-apoptotic effects observed in GPER KO cells. In accordance with these data, survival analyses in TNBC patients of the Molecular Taxonomy of Breast Cancer International Consortium (METABRIC) dataset indicated that high Noxa expression correlates with improved outcomes in TNBC patients. Furthermore, we demonstrated that GPER KO in TNBC cells impairs the expression and secretion of the well-acknowledged GPER target gene named CTGF, thus resulting in the inhibition of migratory effects in cancer-associated fibroblasts (CAFs). Overall, the present study provides novel mechanistic and biological insights on GPER KO in TNBC cells suggesting that GPER may be considered as a valuable target in comprehensive therapeutic approaches halting TNBC progression.

## Introduction

Triple-negative breast cancer (TNBC) is a molecular subtype of breast cancer (BC) lacking the expression of the estrogen receptor (ER), progesterone receptor (PR), and human epidermal growth factor receptor-2 (HER2) [1]. TNBC accounts for approximately 15% of all BC and is considered a major unmet need due to its aggressive features, early recurrence, and adverse prognosis [1]. Compared to other BC subtypes, TNBC has limited treatment options for its peculiar molecular landscape and biological heterogeneity [2]. Therefore, chemotherapy still remains the main therapeutic approach in TNBC patients [3]. Within the intricate transduction network harbored in TNBC cells, diverse receptors involved in estrogen signaling such as ERβ, estrogen-related receptors (ERRs), and the G protein-coupled estrogen receptor (GPER) have been detected [4–6]. In the framework of these studies, previous evidence has indicated that GPER may play a role in TNBC growth and progression [5–8]. GPER, which belongs to the seven-transmembrane G protein-coupled receptor (GPCR) family, has been implicated in numerous pathophysiological conditions including diverse types of cancer [9]. The activation of GPER triggers peculiar transduction pathways as the adenylyl cyclase-mediated production of cAMP, the intracellular calcium mobilization, the mitogen-activated protein kinase (MAPK) and phosphoinositide 3-kinase (PI3K)/Akt cascade [10–13]. Relying on a specific transcription factor signature, GPER regulates genomic changes that lead to relevant biological effects in both normal and malignant tissues, including BC cells [14]. For instance, GPER signaling prompts in TNBC cells proliferative, migratory, and invasive responses to certain estrogens and antiestrogens that are acknowledged to act as GPER agonists [8, 13, 15–19]. Consistent with the aforementioned evidence, GPER has been found highly expressed in TNBC specimens and positively associated with pro-metastatic pathways, tumor size and stage, high recurrence, and poor patient outcomes [5, 6, 16]. GPER can be also deemed as a mediator of stromal function given that it may induce molecular and biological events in different cellular components of the tumor microenvironment like cancer-associated fibroblasts (CAFs), tumor-associated macrophages, and inflammatory/immune cells [20–23]. Accordingly, GPER is expressed and functionally active in CAFs derived from BC patients toward the expression and release of pro-tumorigenic factors, such as inflammatory cytokines and growth factors [20, 24–26]. Together, these observations suggest that GPER may be considered as a molecular hub facilitating the crosstalk between cancer cells and key components of the tumor microenvironment for the occurrence of malignant features.

The Clustered regularly interspaced short palindromic repeats/CRISPR associated nuclease (CRISPR/Cas) system provides a specific and efficient genome editing tool [27]. CRISPR/Cas9 comprises a single-stranded guide RNA (sgRNA) that directs the Cas9 endonuclease to introduce sequence-specific double-stranded breaks in a target gene [28]. The subsequent DNA repair process leads to desired insertions, deletions or substitutions at the cut site and then changes in the expression of the target gene, including genetic knockdown [29]. CRISPR/Cas9 system is widely used in cancer research to dissect the mechanisms underlying tumor development and progression even by repairing disease-causing mutations or knocking out (KO) specific genes [30]. In this vein, encouraging data on the capability of CRISPR/Cas9 to inhibit tumor cell growth by deleting oncogenes or repairing tumor suppressor genes have been attained, thus highlighting this genome editing system as a potential biotechnological approach for cancer treatment [30–32].

In the present study, we have characterized the molecular paths and the biological effects both in vitro and in vivo of GPER KO obtained by CRISPR/Cas9 in MDA-MB-231 cells, which are well acknowledged as a TNBC model system [33]. We show that GPER KO in MDA-MB-231 cells triggers the JNK/p-c-Jun/p53/Noxa transduction pathway, leading to apoptotic features in these TNBC cells. Moreover, we demonstrate that the loss of GPER prevents the CTGF-relied migratory responses in main components of the tumor microenvironment named CAFs, which were isolated from BC patients and exposed to conditioned medium from GPER KO TNBC cells. Overall, our findings uncover new molecular mechanisms through which GPER may be involved in the progression of TNBC and considered as a valuable target in comprehensive therapeutic strategies in patients affected by this malignancy, although future studies are warranted in order to strengthen the present data.

## Results

### Whole transcriptome analysis reveals that GPER KO leads to an anti-proliferative and pro-apoptotic gene expression profile in TNBC cells

**Fig. 1:**
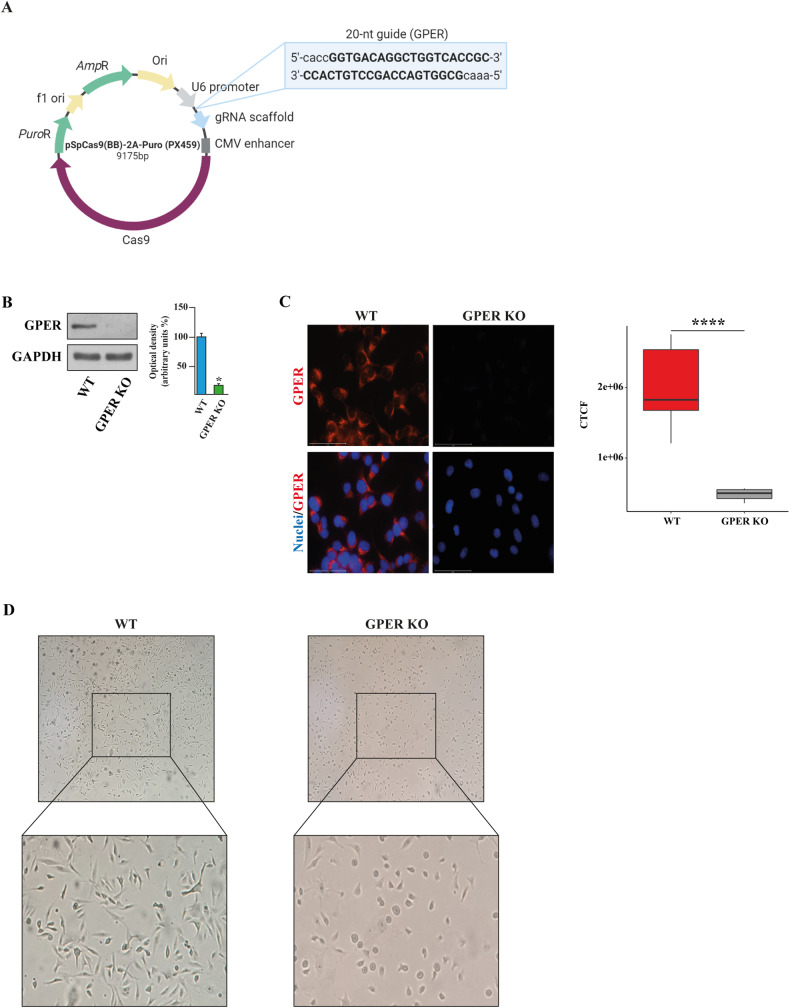
Validation of GPER knockout (KO) in MDA-MB-231 cells. **A** Schematic representation of the PX459 plasmid and the sgRNA sequence used to generate GPER KO MDA-MB-231 cells by CRISPR/Cas9 system. **B** Immunoblots of lysates from WT and GPER KO MDA-MB-231 cells were performed to evaluate the efficiency of GPER KO. Side panels show densitometric analysis of the blots normalized to GAPDH. **C** GPER expression evaluated by immunofluorescence assays in WT and GPER KO MDA-MB-231 cells. Nuclei were stained by DAPI (blue signal). The images shown represent 10 random fields from three independent experiments. Side panel represents corrected total cell fluorescence (CTCF), which was calculated on at least 10 pictures from each sample. Scale bar: 75 μm. Results shown are representative of at least three independent experiments. **D** Morphological appearance of WT and GPER KO MDA-MB-231 cells. *p < 0.05. ****p < 0.0001.

**Fig. 2:**
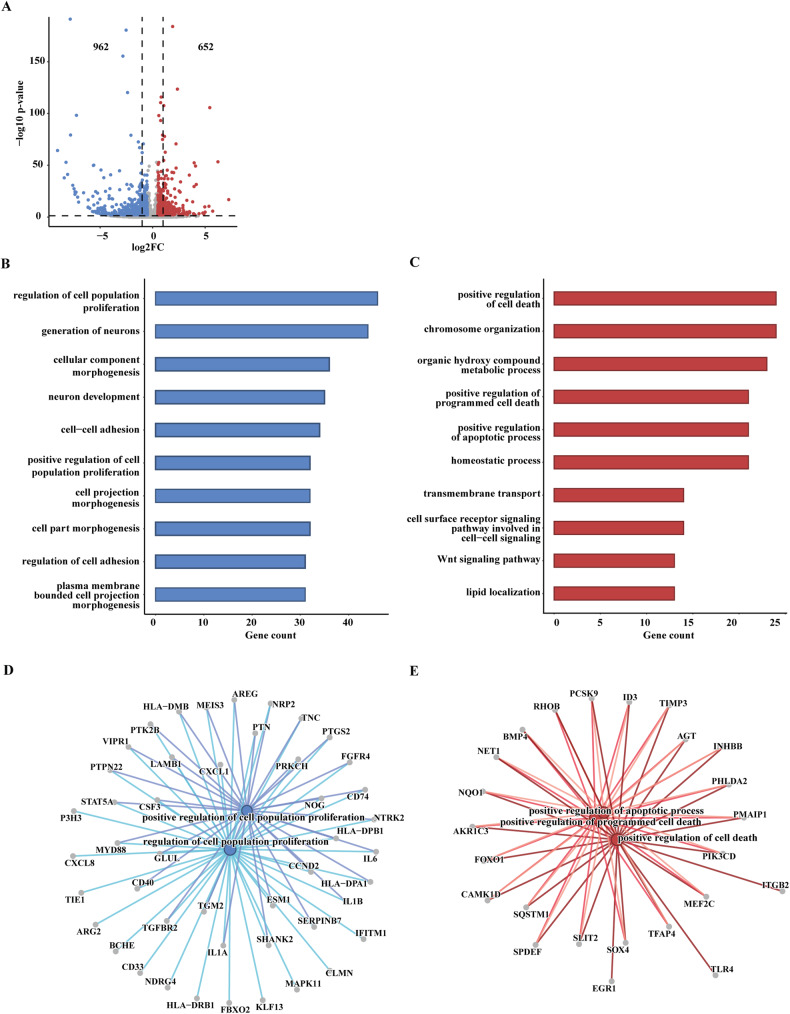
Transcriptomic profiling of WT and GPER KO MDA-MB-231 cells. **A** Volcano plot showing the differentially expressed genes (DEGs) in GPER KO respect to WT MDA-MB-231 cells, as found by transcriptomic analysis. Significantly downregulated genes (log2FC ≤ −0.5 and *p* ≤ 0.01) are shown in blue (n. 962), significantly upregulated genes (log2FC ≥ 0.5 and *p* ≤ 0.01) are shown in red (n. 652), non-significant genes are shown in gray (*p* > 0.01). Gene ontology (GO) analysis of the down- (**B**) and upregulated (**C**) genes in GPER KO respect to WT MDA-MB-231 cells. Bar charts show the top 10 GO terms for biological process (BP) ranked by gene number (high to low). **D** Interrelation analysis of the genes belonging to the “positive regulation of cell population proliferation” and “regulation of cell population proliferation” BPs. **E** Interrelation analysis of the genes belonging to the “positive regulation of cell death”, “positive regulation of programmed cell death” and “positive regulation of apoptotic process” BPs.

Previous studies including our own have indicated that GPER signaling is involved in TNBC progression [4–8]. In order to provide novel insights on the role of GPER in TNBC, we generated a GPER KO MDA-MB-231 cell line by CRISPR/Cas9 genome editing technology (Fig. 1A), as previously described [34]. A matched cell line harboring the empty vector, namely wild-type (WT) MDA-MB-231 cells, was also generated and used as a control. After validating GPER KO efficacy by western blotting and immunofluorescence experiments (Fig. 1B, C), we looked at the morphological changes of GPER KO MDA-MB-231 cells respect to WT MDA-MB-231 cells that are spindle in shape, usually long and flat. The observation by a phase contrast microscope revealed in GPER KO cells a strong increase in roundness compared to WT cells (Fig. 1D). In order to disclose the whole transcriptional changes in GPER KO versus WT MDA-MB-231 cells, we performed high-throughput RNA sequencing (RNA-seq) assessing a total of 1614 differentially expressed genes (DEGs, *p* ≤ 0.01), among which 962 were found downregulated (log2FC ≤ −0.5) and 652 upregulated (log2FC ≥ 0.5) (Fig. 2A). Aiming to unveil the functional role of the identified DEGs, we next performed gene ontology (GO) enrichment analysis. Of note, the downregulated genes were significantly enriched in biological processes (BP) linked to proliferative events (for instance, “positive regulation of cell population proliferation” and “regulation of cell population proliferation”) (Fig. 2B; Supplementary Table 1), whereas the upregulated genes were involved in BP related to cell death and apoptosis (for instance, “positive regulation of apoptotic process”, “positive regulation of programmed cell death” and “positive regulation of cell death”) (Fig. 2C; Supplementary Table 2). The DEGs belonging to the aforementioned BP terms and their interconnections are shown in Fig. 2D, E.

### GPER knockout impairs the growth and suppresses self-renewal in TNBC cells

**Fig. 3:**
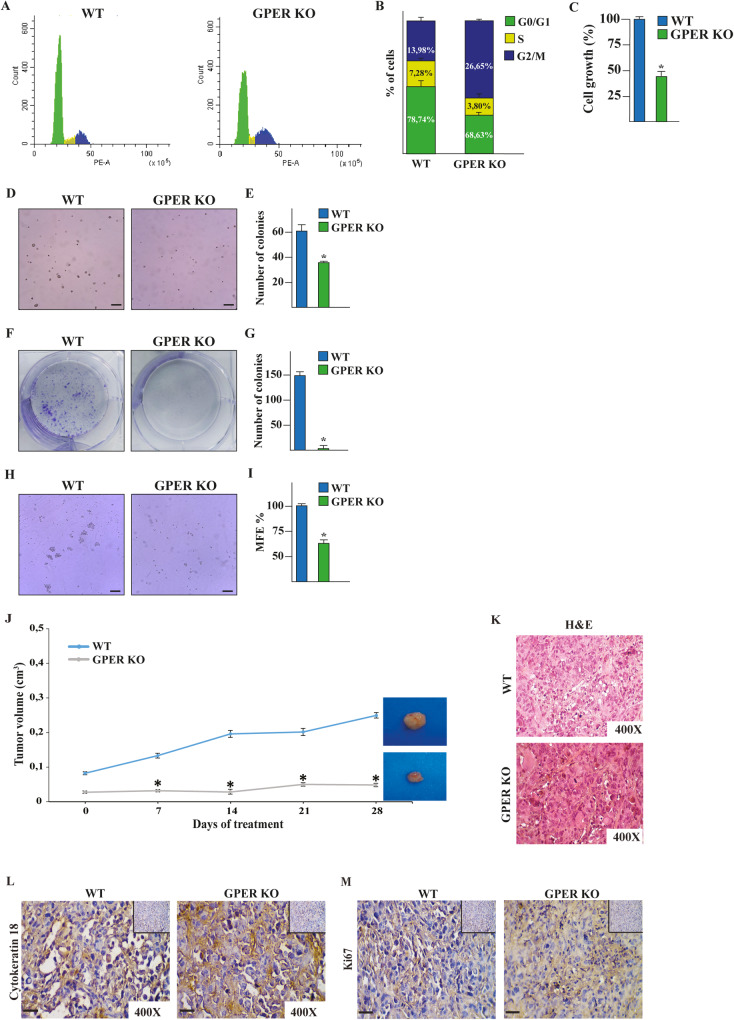
GPER KO prevents TNBC growth in vitro and in vivo. **A** Cell cycle analysis performed by CytoFLEX flow cytometer in WT and GPER KO MDA-MB-231 cells. PE-A, phycoerythrin. **B** Quantitative analysis of percentage gated cells at G0/G1, S, and G2/M phases. **C** Growth of WT and GPER KO MDA-MB-231 cells. Values of WT MDA-MB-231 cells were set as 100% upon which proliferation rate in GPER KO MDA-MB-231 cells was determined. **D** Soft agar-grown colonies of WT and GPER KO MDA-MB-231 cells observed under a microscope on day 20 after seeding. Scale bar: 200 µm. **E** Histogram representation of numbers of colonies. WT and GPER KO MDA-MB-231 cells were cultured in the medium layer of agar and the formation of colonies was captured at 2 weeks after culture. Picture of wells are representative of three independent experiments. **F** Colony formation assay in WT and GPER KO MDA-MB-231 cells. The plates were stained with Giemsa and colonies were counted following 10 days of incubation (**G**). Morphology (**H**) and mammosphere formation efficiency (MFE) (**I**) in second generation mammospheres from WT and GPER KO MDA-MB-231 cells at 20 days of culture. Scale bar: 100 µm. Each column represents the mean ± SD of three independent experiments, each performed in triplicate. **J** Tumor volume from WT and GPER KO MDA-MB-231 xenografts implanted in female athymic nude mice. Tumor growth was monitored by caliper measuring the visible tumor sizes at indicated time points. At the end of experiment (28 day), tumors were explanted, and for each group of animals (*n* = 7), three representative images of tumors are shown. *p < 0.05. **K** Formalin-fixed paraffin-embedded (FFPE) sections of tumor xenografts were stained with hematoxylin and eosin Y (H&E), and the epithelial nature of the tumors was verified by immunostaining with anti-human cytokeratin 18 antibody (**L**). **M** The expression of Ki-67, as a marker of proliferation, was evaluated in FFPE sections of explanted tumors from WT and GPER KO MDA-MB-231 xenografts. Scale bar: 25 µm. Insert: negative control.

On the basis of the aforementioned results, we investigated the biological relevance of GPER KO in MDA-MB-231 cells analyzing the cell cycle regulation, the proliferation, the clonogenic ability, and the stem cell activity. Exploring the cell cycle progression by PI staining, we found a significantly higher percentage of GPER KO in G2/M-phase compared to WT cells along with a concomitant reduction in the number of cells in the S phase (Fig. 3A, B). Furthermore, GPER KO cells showed a reduced proliferative rate in both 2D (Fig. 3C) and 3D soft agar assays (Fig. 3D, E) as well as an impaired colony-forming ability (Fig. 3F, G) respect to WT cells. These findings may suggest that the inhibition of cell growth in GPER KO cells may be associated with a cell cycle arrest in G2/M-phase. Next, we investigated other potential determinants of TNBC aggressiveness exploring the self-renewal capability of the mammosphere-forming MDA-MB-231 cells, which are acknowledged to exhibit certain features associated with mammary cancer stem cells (CSCs) like the CD44^+/high^/CD24^−/low^ phenotype [35]. Of note, GPER KO MDA-MB-231 cells decreased stem cell activity, as argued by their reduced number of secondary mammospheres in comparison with WT MDA-MB-231 cells (Fig. 3H, I). To provide further evidence on the role of GPER in TNBC progression in vivo, we turned to tumor xenograft experiments via orthotopic implantation of WT and GPER KO MDA-MB-231 cells into the mammary fat pad region of 45-day-old female nude mice. Worthy, GPER KO cells developed into smaller tumors respect to WT cells (Fig. 3J, K). Immunostaining positivity for the human cytokeratin 18 revealed the epithelial nature of the tumors (Fig. 3L). Moreover, tumor tissue sections obtained from GPER KO MDA-MB-231 xenografts showed lower levels of the proliferative marker Ki67 respect to WT xenografts (Fig. 3M).

### The cAMP-dependent inhibition of the JNK/p-c-Jun/p53 axis is rescued in GPER KO cells toward intrinsic apoptotic death

**Fig. 4:**
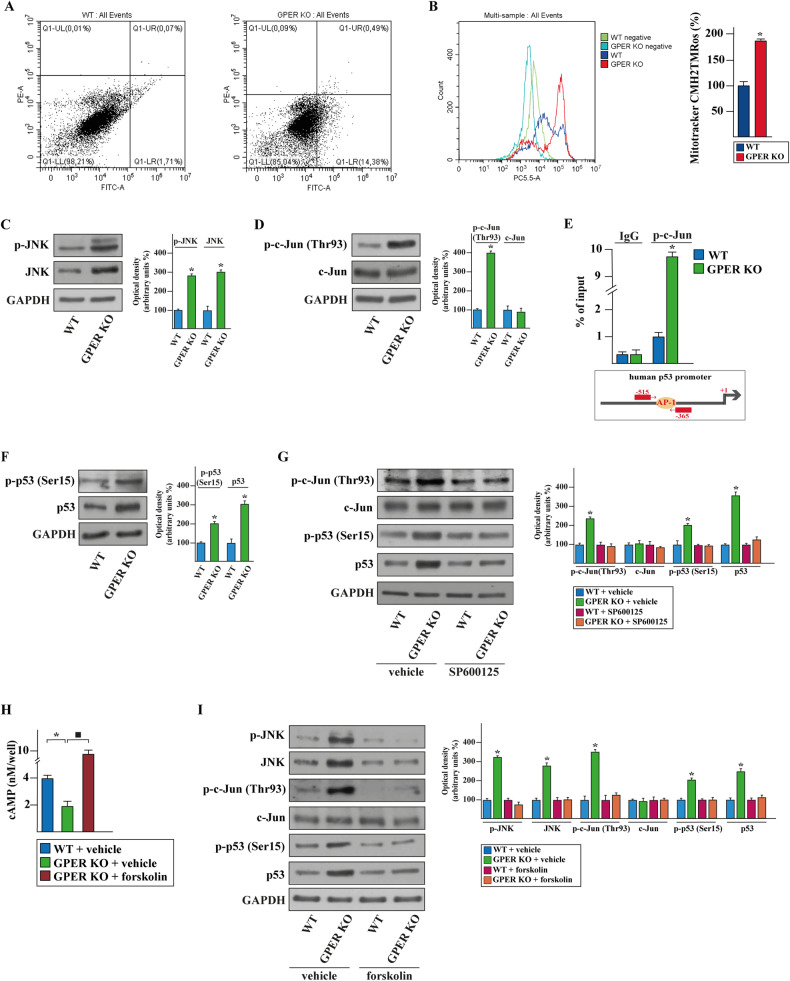
The JNK/p-c-Jun/p53 system is involved in the apoptotic features of GPER KO MDA-MB-231 cells. **A** Cytometric analysis of apoptosis in WT and GPER KO MDA-MB-231 cells. Cells were stained with Annexin V-FITC (Alexa Fluor 488) conjugate to identify apoptotic cells and with propidium iodide (PI) to identify dead cells. The four quadrants represent living cells (lower left, AnnexinV-/PI-), early apoptotic (lower right, Annexin V+/PI-), late apoptosis (upper right, Annexin V+/PI+) or necrotic (upper left, Annexin V-/PI+) stages. Values shown represent the percentages of each quadrant. PE-A, phycoerythrin; FITC-A, fluorescein isothiocyanate-A. **B** Mitochondrial membrane potential in WT and GPER KO MDA-MB-231 cells exposed to MitoTracker Orange CMH2TMRos probe. Side panel represents the mean ± SD of three independent experiments, each performed in triplicate. PC5.5-A, PE-cyanine 5.5-A. Protein levels of p-JNK and JNK (**C**), and p-c-Jun (Thr93) and c-Jun (**D**) in WT and GPER KO MDA-MB-231 cells. **E** Recruitment of p-c-Jun to the AP-1 site located within the p53 promoter sequence in WT and GPER KO MDA-MB-231 cells. In control samples non-specific IgG was used instead of the primary antibody. The amplified sequences were evaluated by real-time PCR. **F** Levels of p-p53 (Ser15) and p53 in WT and GPER KO MDA-MB-231 cells. **G** Protein levels of p-c-Jun (Thr93), c-Jun, p-p53 (Ser15) and p53 in WT and GPER KO MDA-MB-231 cells exposed for 12 h to vehicle or 400 nM JNK inhibitor SP600125. **H** Measurement of cAMP in WT and GPER KO MDA-MB-231 cells exposed for 12 h to vehicle or 10 µM cAMP specific activator forskolin, as indicated. **I** Protein levels of p-JNK, JNK, p-c-Jun (Thr93), c-Jun, p-p53 (Ser15) and p53 in WT and GPER KO MDA-MB-231 cells exposed for 12 h to vehicle or 10 µM forskolin. Side panel shows densitometric analysis of the blots normalized to GAPDH that served as loading control. Results shown are representative of at least three independent experiments. Each column represents the mean ± SD of three independent experiments, each performed in triplicate. (*), (■) indicate *p* < 0.05.

Based on the RNA-seq data and the biological responses described above, the annexin V FITC/PI double staining was used to determine whether GPER deficiency may lead to apoptotic effects in MDA-MB-231 cells. Considering the higher number of apoptotic cells in GPER KO MDA-MB-231 cells observed by flow cytometry analysis (Fig. 4A), we wondered whether the intrinsic pathway may be involved in this response. Disruption of mitochondrial integrity is one of the early events leading to apoptosis [36], therefore we analyzed mitochondrial function using MitoTracker Orange. An increased staining intensity was found in GPER KO respect to WT cells, indicating a reduction in the mitochondrial membrane potential along with an increased mitochondrial outer membrane permeabilization (MOMP) (Fig. 4B). To delineate the underlying molecular mechanism responsible for the aforementioned events, we investigated the expression and activation of Jun N-terminal kinases (JNKs), which are included among the mediators of the mitochondrial intrinsic apoptotic pathway [37]. Interestingly, GPER KO showed upregulated protein levels of both p-JNK and JNK respect to WT MDA-MB-231 cells (Fig. 4C). In this context, it is well recognized the capacity of JNKs to phosphorylate c-Jun that in turn transactivates the AP-1 transcription factor toward the regulation of diverse genes including p53 [38]. Consistent with these observations, in GPER KO but not in MDA-MB-231 WT cells we assessed the activation of c-Jun (Thr93) along with unchanged c-Jun expression levels (Fig. 4D). Moreover, chromatin immunoprecipitation (ChIP) assays revealed that in GPER KO, but not in WT cells, p-c-Jun is recruited to the AP-1 site located within the human p53 promoter sequence (Fig. 4E). These findings were paralleled by increased activation and expression of p53 in GPER KO respect to MDA-MB-231 WT cells (Fig. 4F). Accordingly, the JNK specific inhibitor SP-600125 abrogated in GPER KO cells the phosphorylation of both c-Jun and p53 (Ser15) as well as the increase of p53 expression levels (Fig. 4G). Overall, these data suggested that p-c-Jun, upon activation by p-JNK, triggers the transcriptional regulation of p53 in GPER KO MDA-MB-231 cells.

Then, we attempted to identify the upstream signaling events that may drive the activation of the JNK/p-c-Jun/p53 axis in GPER KO MDA-MB-231 cells. Similar to other GPCRs, the signal transduction mediated by GPER may rely on the production of cyclic AMP (cAMP) and the subsequent activation of the PKA signaling pathway [12, 39]. Considering that cAMP detection may constitute an important readout for monitoring GPCR activation and reminiscing previous reports on the ability of the cAMP/PKA axis to inhibit JNK activation and to suppress apoptosis [40, 41], we demonstrated that GPER KO leads to a reduced production of cAMP levels (Fig. 4H). Worthy, the cAMP pathway activator forskolin restored the cAMP levels (Fig. 4H) and abrogated the upregulation of p-JNK, JNK, p-c-Jun, p-p53 and p53 observed in GPER KO MDA-MB-231 cells (Fig. 4I). Altogether, these results show that the cAMP-dependent attenuation of JNK/p-c-Jun/p53 cascade is lost in GPER KO MDA-MB-231 cells.

### The JNK/p-c-Jun/p53/Noxa axis contributes to the apoptotic features of GPER KO TNBC cells

**Fig. 5:**
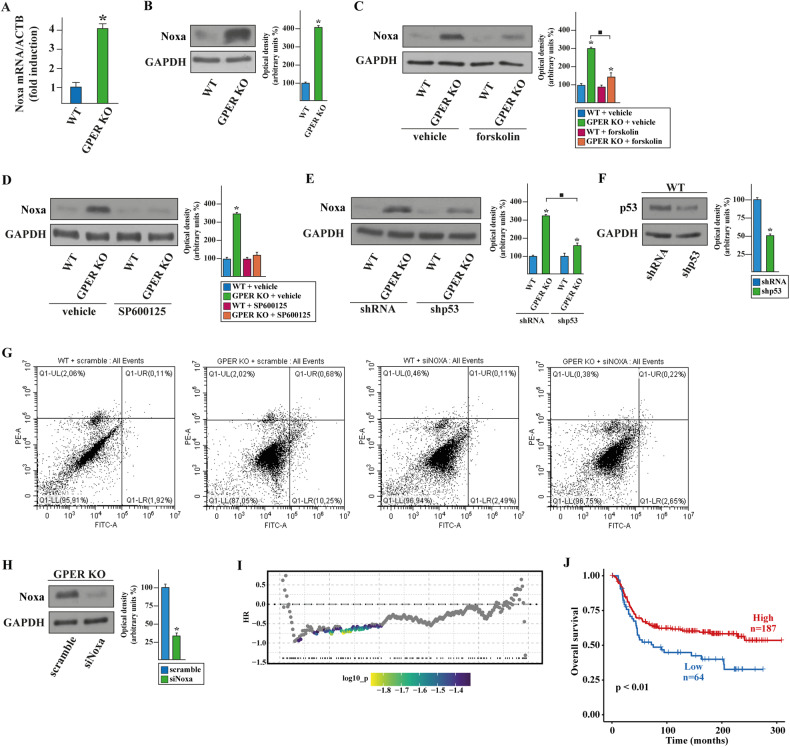
Noxa mediates the mitochondrial apoptotic death in GPER KO MDA-MB-231 cells. mRNA (**A**) and protein (**B**) levels of Noxa evaluated respectively by real-time PCR and immunoblotting in WT and GPER KO MDA-MB-231 breast cancer cells. In RNA experiments, values are normalized to the actin beta (ACTB) expression and presented as fold changes of mRNA expression upon GPER KO relative to WT. **C** Immunoblots of Noxa in WT and GPER KO MDA-MB-231 cells exposed for 12 h to vehicle or 10 µM cAMP activator forskolin. **D** Protein levels of Noxa in WT and GPER KO MDA-MB-231 cells exposed for 12 h to vehicle or 400 nM JNK inhibitor SP600125. **E** Protein levels of Noxa in WT and GPER KO MDA-MB-231 cells transfected for 36 h with shRNA or shp53. **F** Efficacy of p53 silencing. **G** Cytometric analysis of apoptosis in WT and GPER KO MDA-MB-231 cells transfected for 36 h with scramble or siNoxa, as indicated. Cells were stained with Annexin V-FITC (Alexa Fluor 488) conjugate to identify apoptotic cells and with propidium iodide (PI) to identify dead cells. The four quadrants represent living cells (lower left, AnnexinV-/PI-), early apoptotic (lower right, Annexin V+/PI-), late apoptosis (upper right, Annexin V+/PI+) or necrotic (upper left, Annexin V-/PI+) stages. Values represent the percentages of each quadrant. PE-A, phycoerythrin; FITC-A, fluorescein isothiocyanate-A. **H** Efficacy of Noxa silencing. Side panels show densitometric analysis of the blots normalized to GAPDH that served as loading control. Results shown are representative of at least three independent experiments. (*), (■) indicate *p* < 0.05. **I** survivALL plot depicting the TNBC patients of the METABRIC cohort ordered by increasing expression of Noxa (*x*-axis). Hazard ratios (HR) for all possible cut-points to be examined are shown on the *y*-axis. The color bar gradient indicates the range of the most significant points-of-separation of the population (low-high significance = blue-yellow gradient) based on Noxa expression and overall survival (OS) of each patient. **J** Kaplan–Meier plot showing the correlation between Noxa mRNA expression and OS of the METABRIC cohort of TNBC.

The stress-induced apoptosis mediated by p53 triggers the B-cell lymphoma 2 (Bcl-2) family members, including the BH3-only protein namely Noxa, which is regulated by the JNK signaling [42–44]. Thus, we asked whether the activation of JNK/p-c-Jun/p53 cascade might regulate Noxa expression and activity in GPER KO MDA-MB-231 cells. As determined by RNA-seq data, the mRNA (Fig. 5A) and protein (Fig. 5B) levels of Noxa are higher in GPER KO than in WT MDA-MB-231 cells. Thereafter, we ascertained that the upregulation of Noxa protein levels is prevented by the cAMP activator forskolin (Fig. 5C), the JNK inhibitor SP600125 (Fig. 5D), and silencing p53 expression (Fig. 5E, F). Hence, we sought to examine the role of Noxa in the apoptosis of GPER KO MDA-MB-231 cells by performing Annexin V FITC/PI assay. Noxa silencing by small interfering RNA (siRNA) technology completely abolished the increased apoptotic rate caused by GPER deficiency (Fig. 5G, H), clearly suggesting the involvement of Noxa in the apoptosis of GPER KO cells. Taken together, these findings indicate that GPER loss in TNBC cells induces pro-apoptotic events mainly through the decrease of cAMP levels and the ensuing stimulation of the JNK/p-c-Jun/p53/Noxa axis. In order to corroborate the clinical implications for Noxa expression in TNBCs, samples of the METABRIC cohort were ranked in accordance with the differential expression of Noxa (low to high) and all the possible cut-points of the population were calculated, as shown in the survivALL plot (Fig. 5I). Considering the most significant cut-point, the related Kaplan–Meier survival curves indicated that a better overall survival (OS) characterizes TNBC patients displaying high Noxa expression (Fig. 5J). In agreement with these findings, low Noxa levels were found in BC patients with stage III (Supplementary Fig. 1A) and histological G3 (Supplementary Fig. 1B). Further supporting these data, high Noxa expression was assessed to correlate with a good prognosis in BC patients according to the NPI classification (Supplementary Fig. 1C) as well as with low invasive BC subtypes (Supplementary Fig. 1D).

### GPER deficiency in TNBC cells prevents the CTGF-dependent migratory effects in CAFs

**Fig. 6:**
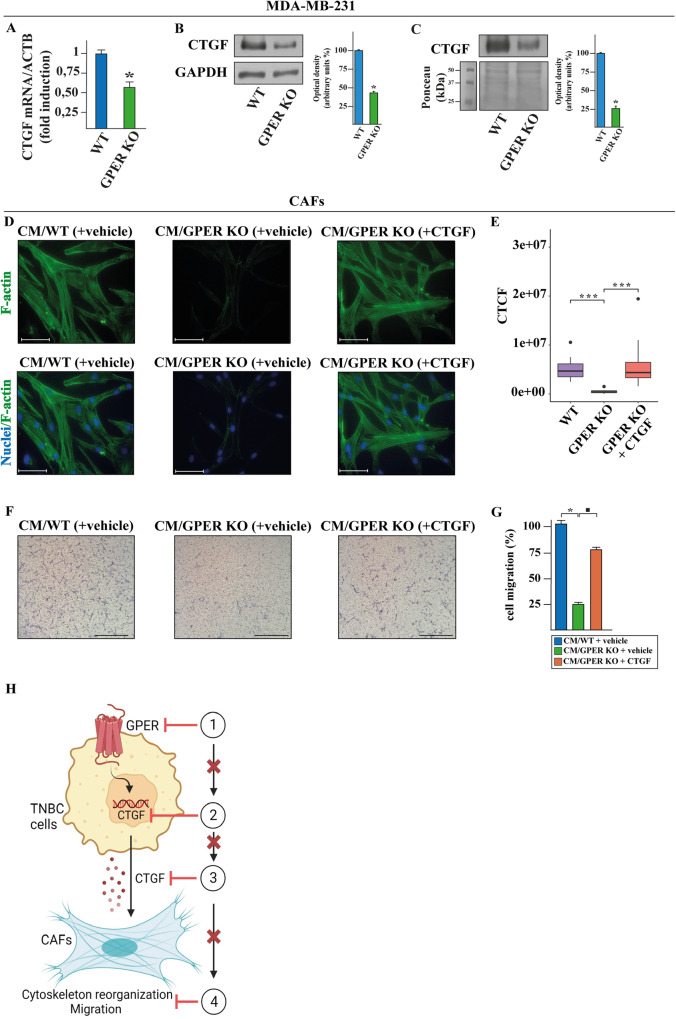
GPER KO in MDA-MB-231 cells suppresses the CTGF-driven cytoskeleton reorganization and migration in CAFs. mRNA (**A**) and protein (**B**) levels of CTGF evaluated respectively by real-time PCR and immunoblotting in WT and GPER KO MDA-MB-231 cells. In RNA experiments, values are normalized to the actin beta (ACTB) expression and presented as fold changes of mRNA expression upon GPER KO relative to WT. **C** Evaluation by immunoblotting of CTGF protein levels in conditioned medium (CM) collected from WT and GPER KO MDA-MB-231 cells. Ponceau red staining of the membrane was used as a loading control for the CM. Side panels show densitometric analysis of the blots normalized to GAPDH that served as loading control. **D** FITC-phalloidin staining of CAFs cultured for 12 h with conditioned medium (CM) collected from WT and GPER KO MDA-MB-231 cells, which were previously treated for 24 h with vehicle or 100 nM CTGF, as indicated. Cells were stained with FITC-phalloidin to detect F-actin stress fibers (green) and with DAPI to detect nuclei (blue). The images shown represent 10 random fields from three independent experiments. **E** Corrected total cell fluorescence (CTCF) was calculated on at least 10 pictures from each sample. Scale bar: 75 μm. Results shown are representative of at least three independent experiments. Values represent the mean ± SD of three independent experiments performed in triplicate. **F** Transwell migration assay in CAFs cultured for 12 h with conditioned medium (CM) collected from WT and GPER KO MDA-MB-231 cells, which were previously treated for 24 h with vehicle or 100 nM CTGF, as indicated. **G** Evaluation of cell migration in 10 random fields in each of three independent experiments performed in triplicate. Scale bar: 200 μm. (*) and (■) indicate *p* < 0.05. (***) indicate (*p* < 0.001). **H** Cartoon depicting the effects of GPER KO (1) in inhibiting the expression of CTGF (2) and its secretion (3) by MDA-MB-231 cells, thus preventing F-actin cytoskeleton reorganization and migratory effects (4) in CAFs. Created with BioRender.com.

GPER signaling promotes migration of BC cells through the induction of the connective tissue growth factor (CTGF) [45]. On these bases and considering that CTGF is involved in the stimulation of the tumor microenvironment, including CAFs activation [46], we verified whether the GPER-mediated release of CTGF from MDA-MB-231 cells can trigger motile responses in BC-derived CAFs. First, by real-time PCR (Fig. 6A) and immunoblotting (Fig. 6B) experiments we ascertained that the expression of CTGF is lower in GPER KO respect to WT MDA-MB-231 cells. Then, we found that CTGF levels are downregulated in conditioned medium (CM) of GPER KO than WT MDA-MB-231 cells (Fig. 6C). To explore whether the GPER-dependent increase of CTGF can prompt stimulatory responses in CAFs, we evaluated the potential of conditioned medium (CM) collected from GPER KO and WT MDA-MB-231 cells to induce migration in CAFs. First, we observed a reduction in phalloidin-labeled actin stress fibers in CAFs cultured with CM from GPER KO MDA-MB-231 cells respect to CAFs exposed to CM from WT MDA-MB-231 cells (Fig. 6D, E). Worthy, these events were restored by adding CTGF (Fig. 6D, E). Then, we assessed that the reduced migratory ability of CAFs exposed to the CM from GPER KO respect to CAFs exposed to CM from WT MDA-MB-231 cells was rescued by adding CTGF (Fig. 6F, G). Overall, these data suggest that GPER KO MDA-MB-231 cells are characterized by dampened CTGF expression and release leading to decreased migratory properties of CAFs (Fig. 6H).

## Discussion

GPER is a seven-transmembrane receptor belonging to the GPCRs family [39]. Independently of the classical estrogen receptors (ERα and ERβ), GPER mediates estrogen signaling in different normal and malignant tissues [47]. Intracellular signal events initiated by GPER occur via coupling with either Gα or Gβγ subunits and may involve the transactivation of the epidermal growth factor receptor (EGFR) [47]. Arising upon stimulation by estrogen and estrogen-like compounds, the activation of GPER triggers the mitogen-activated protein kinases (MAPK) ERK1/2, PI3K/Akt, phospholipase C, adenylyl cyclase and cAMP production [13]. In addition, GPER prompts a peculiar gene expression profile, which includes c-Fos and CTGF, toward relevant biological effects like proliferation, survival, migration, invasion, tumor-associated angiogenesis, and inflammation [13, 45]. As it concerns BC, the expression of GPER is correlated with clinicopathological indices that predict disease progression and poor survival [48, 49]. Likewise, GPER is an independent prognostic factor for decreased disease-free survival in BC patients treated with tamoxifen [50]. Consistent with these findings, both in vitro and in vivo studies have shown the involvement of GPER in BC cell growth, migration and invasion, in mammary tumorigenesis and metastasis as well as in tamoxifen resistance [18, 26, 45, 51–54].

A role of GPER has been also suggested in the progression of TNBC, which is responsive to estrogen stimulation albeit it lacks the classic ERs. For instance, the activation of GPER triggers pro-tumorigenic transduction pathways like focal adhesion kinase (FAK) and ERK1/2 signaling, toward the stimulation of growth, migration and invasion of TNBC cells [8, 15–19]. Of note, the hypoxia-induced cooperation between GPER and interleukin-1β (IL-β) axis generates a metastatic gene signature that leads to proliferative and invasive effects in TNBC cells [18]. Corroborating these findings, high expression levels of GPER correlate with poor outcome in premenopausal BC patients in terms of overall survival (OS), progression-free survival (PFS), local relapse-free survival (LRFS) and distant disease-free survival (DDFS) [7]. Besides, high expression of GPER is associated with pro-metastatic pathways and worse survival in patients with advanced TNBCs [6]. Nevertheless, other studies suggested a tumor-suppression action of GPER in TNBC, therefore its role in the context of the biological TNBC landscape remains to be fully explored [55–58]. This controversial scenario prompted us to generate GPER KO TNBC cells through the CRISPR-Cas9 gene editing system, which is widely used in order to contribute to unveil cancer genomics [59–61]. Whole-transcriptome sequencing, complemented with GO enrichment analysis, revealed that GPER KO leads to a diminished transcription of genes involved in cell proliferation. Accordingly, we found that GPER KO MDA-MB-231 cells display a lower proliferative rate compared with the normal counterpart either in vitro or in vivo xenograft models. Moreover, the loss of GPER contributed to the reduction of the stem-like phenotype of MDA-MB-231 cells. Moreover, we demonstrated that GPER deficiency is associated with an increased apoptotic death, in accordance with the RNA-seq data showing the upregulation of apoptotic-related genes in GPER KO respect to WT MDA-MB-231 cells. In particular, we found that GPER KO dependent apoptosis relies on the mitochondria-mediated death pathway, as determined through the cytometric assessment of mitochondria function.

**Fig. 7:**
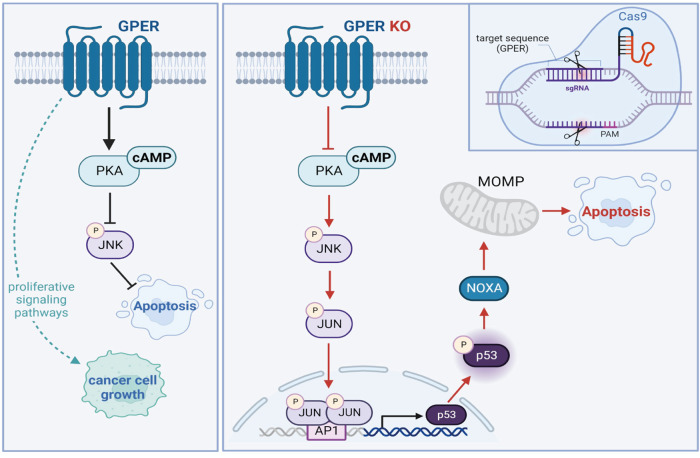
Molecular paths engaged by CRISPR/Cas9-mediated GPER KO leading to apoptotic death in MDA-MB-231 TNBC cells. Created with BioRender.com.

The intrinsic apoptotic pathway is regulated by the integrity of mitochondria at both structural and functional level [36, 62]. The Bcl-2 family, which consists of members with opposite functions, orchestrates mitochondrial outer membrane permeabilization (MOMP) [63]. The balance among diverse Bcl-2 members regulates cell fate by inducing or preventing the release into the cytosol of pro-apoptotic proteins from the mitochondrial intermembrane space [64, 65]. Importantly, the tumor suppressor gene p53 exerts a critical role in the transcription of Bcl-2 family members like Bax and the BH3-only (Bcl-2 homology 3) proteins Bid, Noxa and PUMA [66]. The Jun N-terminal kinases (JNKs) play a critical role in mitochondrial intrinsic apoptotic pathway. In particular, JNKs activate apoptotic signals inducing pro-apoptotic genes via the phosphorylation and transactivation of c-Jun, which is a major component of the dimeric transcription factor AP-1 [67, 68]. Of note, the JNK/c-Jun/AP-1 axis is involved in the downregulation of pro-survival genes as well as in the upregulation of transcription factors and pro-apoptotic genes as p53 [69]. In addition, JNKs mediate apoptosis activating proteins of the p53 family and inhibiting their ubiquitin-mediated degradation [70]. In this scenario, we have demonstrated a novel mechanism by which the JNK signaling pathway triggers the cell death machinery in GPER KO TNBC cells. Indeed, we have found that JNK activation stimulates through the c-Jun/AP-1 pathway the expression and phosphorylation of p53, which in turn leads to the regulation of the pro-apoptotic protein Noxa. In particular, in GPER KO MDA-MB-231 cells we ascertained that JNK signaling is required for c-Jun phosphorylation and its recruitment to the AP-1 site located within the p53 promoter sequence, toward the induction of a main mediator of p53-dependent apoptosis namely Noxa (Fig. 7). Consistent with previous findings showing that the levels of Noxa are associated with improved relapse-free survival (RFS) and overall survival (OS) in BC patients and increase upon p53 overexpression [43, 71], our analysis of the METABRIC cohort of patients showed that high Noxa levels are predictive for a better outcome in TNBC patients.

In order to further dissect the mechanisms by which JNK signaling primes the p53/Noxa-dependent apoptotic death in GPER KO MDA-MB-231 cells, we have also explored the involvement of cAMP in these effects on the basis of previous evidence reporting that the cAMP/PKA pathway may inhibit JNK activation [40]. Ligand-induced signal transduction via specific classes of GPCRs can rely upon the production of cAMP as second messenger [39]. However, GPCRs can produce a baseline level of cAMP even in a ligand-independent manner [72]. In this context, we have found that GPER KO leads to decreased cAMP levels and the consequent upregulation of the JNK pathway, which in turn stimulates the p-c-Jun/p53/Noxa axis (Fig. 7), as determined by the use of the AC/cAMP activator forskolin in GPER KO MDA-MB-231 cells.

Connective tissue growth factor (CTGF) belongs to the CCN family of secreted matrix-associated proteins that mediate cell differentiation, proliferation, chemotaxis, migration, adhesion, and angiogenesis interacting with diverse cell surface proteins [73]. In addition, CTGF may promote tumor progression, metastasis, and drug resistance in BC as well as in pancreas, prostate and brain tumors [74]. Of note, the correlation between CTGF expression and poor prognosis in TNBC patients has been recently demonstrated [75]. Accordingly, it has been found that CTGF is overexpressed in TNBC cells and mediates the migration and invasion through the integrin αvβ3/ERK pathway as well as aerobic glycolysis via the FAK/Src/NF-κB/Glut3 axis [75, 76]. Reminiscing these observations and in line with our previous microarray transcriptome analysis showing that CTGF is as a main target gene of GPER [45], here we have assessed that GPER KO MDA-MB-231 cells display a reduced expression and secretion of CTGF.

It is well recognized that cancer progression relies not only on neoplastic cells, but also on their functional crosstalk with the surrounding stroma [77–79]. The interplay between cancer cells and tumor microenvironment (TME) components is bidirectional and dynamic as it may occur through direct cell-cell contacts and/or soluble molecules that mediate non-physical communications among components of the TME and cancer cells. Furthermore, CAFs stimulate cancer cell proliferation, angiogenesis, migration, invasion and metastatic spreading inducing epithelial to mesenchymal cell transition (EMT) and in parallel tumor cells can initiate and sustain CAFs activation [77, 78]. Several studies have shown that CAFs co-migrate with tumor cells in the bloodstream, suggesting that CAFs activation may be required for the formation of the pre-metastatic niches [80, 81]. Both tumor cells and CAFs themselves generate stimuli and signaling pathways that promote CAFs motility [82, 83]. In this regard, we have shown that the GPER-mediated release of CTGF from MDA-MB-231 cells triggers the reorganization of the actin cytoskeleton toward the migration of CAFs obtained from BC patients. Nicely fitting with these results, we have found that the aforementioned effects are abolished knocking out GPER expression in MDA-MB-231 cells. These data suggest that CTGF may be considered as a mediator of the migratory effects sustained by GPER at the crossroad between TNBC cells and CAFs (Fig. 6H).

Our findings provide novel mechanistic insights for a better comprehension on the role elicited by GPER in TNBC. In particular, we have demonstrated that GPER KO leads to an anti-proliferative and pro-apoptotic gene expression pattern in TNBC cells. Our data have also addressed the new signaling network relying on the JNK/c-Jun/p53/Noxa axis as an orchestrator of a pro-apoptotic behavior of GPER KO TNBC cells (Fig. 7). Furthermore, we have determined that GPER triggers CTGF expression and release in TNBC cells toward the migratory phenotype of CAFs. Overall, our results imply that the genetic suppression of GPER might be considered for more comprehensive therapeutic strategies halting TNBC progression, although further studies are warranted to strengthen the present data.

## Materials and methods

### Reagents

SP600125 (SP) was purchased from MedChemExpress (DBA, Milan, Italy), Forskolin from Merck Life Science (Milan, Italy) and CTGF from Thermo Fisher Scientific (Milan, Italy). The compounds were solubilized in dimethyl sulfoxide (DMSO), ethanol and water respectively.

### Cell Cultures

MDA-MB-231 cells were obtained from the ATCC (Manassas, USA) and were maintained in DMEM/F12 (Thermo Fisher Scientific, Milan, Italy), 5% fetal bovine serum (FBS) and 1% of penicillin/streptomycin. Cells were used <6 months after resuscitation and mycoplasma negativity was tested monthly. CAFs were obtained from 10 invasive ductal breast carcinomas and pooled for the subsequent studies, as previously described [84]. Briefly, specimens were cut into 1–2 mm diameter pieces, placed in a digestion solution comprising 400 IU collagenase, 100 IU hyaluronidase, 10% serum, antibiotics, and antimycotics, and incubated overnight at 37 °C. After centrifugation at 90 × *g* for 2 min, the supernatant containing fibroblasts was centrifuged at 485 × *g* for 8 min; the pellet obtained was suspended in Medium 199 and Ham’s F12 mixed 1:1 (supplemented with 10% FBS and 100 μg/ml penicillin/streptomycin). CAFs were then expanded into 10-cm Petri dishes and stored as cells passaged for three population doublings within a total of 7 to tissue dissociation. Primary cultures of fibroblasts were characterized by immunofluorescence, as previously described [84]. We used CAFs passaged for up to 10 population doublings for the experiments to minimize clonal selection and culture stress, which could occur during extended tissue culture. All cells were grown in a 37 °C incubator with 5% CO_2_ and switched to 1% charcoal-stripped FBS for 36 h to be processed for experiments.

### CRISPR/Cas9-mediated GPER knockout (KO)

Short guide RNA (sgRNA) sequence targeting human GPER was designed using the E-CRISP sgRNA Designer (http://www.e-crisp.org/E-CRISP/) and cloned into the pSpCas9 (BB)-2A-Puro (PX459) vector (kindly provided by Dr. W.T. Khaled, University of Cambridge, UK) according to the protocol described in Ran et al. [85]. The GPER sgRNA sequence is as follows: sgGPER: 5′-GGTGACAGGCTGGTCACCGC-3′. Then, the plasmid with sgRNA was transiently transfected into MDA-MB-231 cells using Lipofectamine LTX (L Thermo Fisher Scientific, Milan, Italy). Two days after transfection, the cells were selected via growth in a medium containing 1 µg/mL puromycin dihydrochloride (Merck Life Science, Milan, Italy). After puromycin selection, the puromycin-resistant colonies were picked and expanded in regular medium. Immunoblots and immunofluorescence assays were then performed to evaluate the efficiency of the GPER knockout.

### Conditioned medium

WT and GPER KO MDA-MB-231 cells were placed in 1% charcoal-stripped FBS and treated with vehicle or 100 nM CTGF for 24 h. The supernatants were then collected, centrifuged at 3500 rpm for 5 min to remove cell debris and used as conditioned medium in the appropriate experiments.

### Gene expression studies

Total RNA was extracted and cDNA was synthesized by reverse transcription as described in our previous work [18]. The expression of selected genes was quantified by real-time PCR using platform Quant Studio7 Flex Real-Time PCR System (Thermo Fisher Scientific, Milan, Italy). Gene-specific primers were designed using Primer Express version 2.0 software (Applied Biosystems) and are as follows: 5′-AAGCCACCCCACTTCTCTCTAA-3′ (ACTB forward) and 5′-CACCTCCCCTGTGTGGACTT-3′ (ACTB reverse); 5′-ACCTGTGGGATGGGCATCT-3′ (CTGF forward) and 5′-CAGGCGGCTCTGCTTCTCTA-3′ (CTGF reverse), 5′-GAGGAACAAGTGCAAGTAGCTG-3′ (Noxa forward) and 5′-GGAGTCCCCTCATGCAAGTT-3′ (Noxa reverse). Assays were performed in triplicate and the results were normalized for actin beta (ACTB) expression and then calculated as fold induction of RNA expression.

### RNA-Seq pipeline

RNA integrity for library preparation was determined by analysis of extracted total RNA using a 2100 Bioanalyzer (Agilent Technologies) with RNA 6000 NanoChip. RNA concentrations were measured using Qubit RNA Assay Kit. Libraries were prepared from total RNA according to manufacturer instructions with Illumina Stranded mRNA Prep kit. Libraries quality were evaluated by size analysis on 2100 Bioanlyzer (Chip DNA HS) and concentrations were determined using Qubit DNA HS assay kit (Thermo Fisher). Sequencing was performed on Illumina Novaseq 6000 in the 100PE format. Reads preprocessing was performed by using fastp v0.20.0 [86], applying specific parameters in order to remove residual adapter sequences and to keep only high-quality data (qualified_quality_phred=20, unqualified_percent_limit=30, average_qual=25, low_complexity_filter=True, complexity_threshold=30). The percentage of uniquely mapped reads resulted high with the mean value of 84% (mean value for sample: 60 million total reads, unmapped reads 7%, quality base >q30 94%). Then, passing filter reads were mapped to the human genome reference (version GRCh38) using STAR v2.7.0 [87] with standard parameters, except for sjdbOverhang option set on read length. Genome and transcripts annotation provided as input were downloaded from v99 of Ensembl repository. Alignments were then elaborated by RSEM v1.3.3 [88], to estimate transcript and gene abundances. Subsequently, the sample-specific gene-level abundances were merged into a single raw expression matrix applying a dedicated RSEM command (rsem-generate-data-matrix). Genes with at least 10 counts in 50% of samples were then selected. Differential expression (pairwise comparisons) was computed by edgeR [89] from raw counts in each comparison, following authors’ instructions. Multiple testing controlling procedure was applied and genes with logFC > |0.5| were considered differentially expressed. Re-annotation of previously differential expressed genes was performed using the bioMart package [90] into R 3.6, querying available Ensembl Gene IDs and retrieving Gene Names and Entrez gene IDs.

### Data collection and bioinformatics analyses

Bioinformatics analyses were performed on R Studio (version 4.1.3). Clinical information and gene expression (Log2 transformed intensity values) of the Molecular Taxonomy of Breast Cancer International Consortium (METABRIC) dataset were retrieved from cBioPortal for Cancer Genomics (http://www.cbioportal.org/) on the 19th of February, 2023. Patients (n. 2509) were classified on the basis of the presence or the absence of the estrogen receptor (ER), progesterone receptor (PR) and human epidermal growth factor receptor 2 (HER2), detected by immunohistochemistry. Gene expression and clinical information were also filtered for missing values. Comprehensive survival analysis was conducted using METABRIC gene expression data of Noxa along with the overall survival (OS) information. Samples were filtered for the vital status and patients classified as “died of other causes” were excluded. The survivALL package was employed to examine Cox proportional hazards for all possible points-of-separation (low-high cut-points) [91], selecting the cut-point with the lowest p-value and separating the patients into high and low Noxa expression levels. The Kaplan–Meier survival curves were generated using the survival and the survminer packages, a log-rank test was used to determine differences between the survival curves. *p* < 0.01 was considered statistically significant. Box plots were performed with the tidyverse package and the related statistical analysis was performed using the Wilcoxon test. For the Nottingham prognostic index (NPI) analysis, patients were grouped into three categories: poor (NPI ≤ 3.4), moderate (NPI > 3.4 but ≤5.4) and good (NPI > 5.4).

### Gene ontology enrichment analysis

In order to explore the biological role of the DEGs resulting from RNA-seq analysis, gene ontology (GO) analysis was performed employing the gseGO() function of the topGO package [92] in R Studio. *P*-value < 0.05 was considered as a significant threshold.

### Gene silencing experiments

Cells were plated onto 10-cm dishes and transfected by X-treme GENE 9 DNA Transfection Reagent for 36 h with a short hairpin construct against p53 (shp53) (SHCLND 11212209MN; NM_000546) or a negative control plasmid (shRNA) (Merck Life Science, Milan, Italy). Silencing of Noxa was performed by transiently transfecting the cells for 36 h, using X-treme GENE 9 DNA Transfection Reagent, with a pool of three unique 27mer siRNA duplexes for Noxa (SR321356) targeting-sequences (siNoxa) (5 nM) or a non-targeting scramble control (5 nM) (SR30004) (OriGene Technologies, DBA, Milan, Italy).

### Western blotting analysis

Cells were grown in 10-cm dishes, exposed to treatments where required, and then lysed in 500 μl RIPA buffer with protease inhibitors (1.7 mg/ml aprotinin, 1 mg/ml leupeptin, 200 mmol/l phenylmethylsulfonyl fluoride, 200 mmol/l sodium orthovanadate, and 100 mmol/l sodium fluoride). Samples were then centrifuged at 13,000 rpm for 10 min and protein concentrations were determined using BCA protein assay according to the manufacturer’s instructions (Thermo Fisher Scientific, Milan, Italy). Equal amounts of whole-protein extract were resolved on 10% SDS polyacrylamide gels and transferred to nitrocellulose membranes (Merck Life Science, Milan, Italy), which were probed with primary antibodies against: GAPDH (G-9), c-Jun (G-4), JNK (D-2), p-JNK (G-7), p53 (DO-1) (Santa Cruz Biotechnology, DBA, Milan, Italy), GPER (ab137479) (Abcam, DBA, Milan, Italy), p-c-Jun (Thr93) (2993S), p-p53 (Ser15) (BK92845) (Cell Signaling Technology, Euroclone, Milan, Italy), CTGF (TA806803) (OriGene Technologies, DBA, Milan, Italy) and Noxa (114C307) (Prodotti Gianni, Milan, Italy). Proteins were detected by horseradish peroxidase-linked secondary antibodies (Bio-Rad, Milan, Italy) and then revealed using the chemiluminescent substrate for western blotting Clarity Western ECL Substrate (Bio-Rad, Milan, Italy). Densitometric analysis was performed using the freeware software ImageJ that allowed to quantify the band intensity of the protein of interest with respect to the band intensity of the loading control.

### Acetone precipitation of proteins

Protein precipitation from conditioned medium derived from CAFs was carried out using the precipitation method with acetone [84, 93]. Briefly, four volumes of ice-cold acetone (Merck Life Science, Milan, Italy) were added to one volume of sample. The mixture was vortexed and incubated at −20 °C overnight. This was followed by centrifugation at 10,000 × *g* for 15 min at 4 °C. Then, the supernatant was discarded, the pellet was air dried and then dissolved in 2X Laemmli buffer to be used in the appropriate experiments. In western blot analysis, the protein loading of CM samples was checked by Ponceau red staining [0.1% Ponceau S (w/v) in 5% acetic acid] of the blotted membranes.

### Immunofluorescence microscopy and phalloidin staining

Cells were grown on a cover slip and then fixed in 4% paraformaldehyde in PBS, permeabilized with 0.2% Triton X-100, washed three times with PBS and incubated at 4 °C overnight with the primary antibody anti-GPER (ab137479) (Abcam, DBA, Milan, Italy). After incubation, the slides were extensively washed with PBS, probed with Alexa Fluor conjugated secondary antibodies (Thermo Fisher Scientific, Milan, Italy) for 1 h at room temperature. Finally, cells were washed three times with PBS, incubated with DAPI (4′,6-diamidino-2-phenylindole) (1:10,000) for 3 min and, after washing, immunofluorescence images were obtained using the Cytation 3 Cell Imaging Multimode reader (BioTek, AHSI, Milan Italy). For phalloidin staining, cells were exposed to conditioned medium (CM) derived from WT and GPER KO MDA-MB-231 cells, washed twice with PBS, fixed in 4% paraformaldehyde in PBS for 10 min, washed briefly with PBS and then incubated with Phalloidin-Fluorescent Conjugate (ab176753) (Abcam, DBA, Milan, Italy). The images were obtained using the EVOSTM M7000 Imaging System (Thermo Fisher Scientific, Milan, Italy). Immunofluorescence images and phalloidin staining images were analyzed using ImageJ software by selecting one cell at the time in each picture and measuring the area, integrated density and mean gray value. Thereafter, the corrected total cell fluorescence (CTCF) = integrated density – (area of selected cell × mean fluorescence of background readings) was calculated. Box plots and statistical analysis (*t*-test) were performed using R-studio.

### Chromatin immunoprecipitation (ChIP) assay

Cells were grown in 10-cm dishes, exposed to treatments and then cross-linked with 1% formaldehyde and sonicated. Supernatants were immunocleared with salmon DNA/protein A-agarose (Merck Life Science, Milan, Italy) and immunoprecipitated with anti-p-c-Jun (Thr93) antibody or nonspecific IgG. Pellets were washed, eluted with a buffer consisting of 1%SDS and 0.1 mol/L NaHCO3, and digested with proteinase K. DNA was obtained by phenol/chloroform extractions and precipitated with ethanol. The yield of target region DNA in each sample after ChIP was analyzed by real-time PCR. The primers used to amplify a region containing an AP-1 site located into the p53 promoter sequence were: 5′-GCAGCCATTCTTTTCCTGCT-3′ (Fw) and 5′-CAGTGACCCGGAAGGCAGTC-3′ (Rv), as indicated. Data were normalized to the input for the immunoprecipitation and the results were reported as fold changes respect to nonspecific IgG.

### Annexin V/propidium iodide (PI) assay

The Annexin V-FITC Kit-Apotosis Detection (Beckman-Coulter, Milan, Italy) was used to perform the annexin V/PI assay. WT and GPER KO MDA-MB-231 cells (2 × 10^5^ /well in 2 ml of medium) were seeded in six-well plates and transfected as previously described, where required. Then the cells were collected with trypsin, centrifuged at 1000/1200 rpm for 5 min, resuspended in PBS, and counted. Cells (1 × 10^6^ cells/ml of buffer) were resuspended in 1× binding buffer provided by the kit. 100 µl (containing 105 cells) were transferred into a tube and incubated with 2 µl of Annexin V and 2 µl of PI for 15 min in the dark at room temperature. At the end of the incubation, 400 µl of Binding Buffer (1X) were added to each tube and the samples were analyzed by CytoFLEX flow cytometry (Beckman-Coulter, Milan, Italy).

### Mitochondrial membrane potential assay

Mitochondrial membrane potential was measured by FACS analysis of cells stained with MitoTracker® Orange CMH2TMRos (mitochondrial membrane potential evaluation) (Thermo Fisher Scientific, Milan, Italy). WT and GPER KO MDA-MB-231 cells were plated and after seeding they were switched to 1% charcoal-stripped FBS for 36 h to be processed for experiments. Next, the cells were collected and incubated with 10 nM MitoTracker staining solution for 30–60 min at 37 °C. Cells were then harvested, re-suspended in PBS and analyzed by flow cytometry (CytoFLEX Beckman, Beckman-Coulter, Milan, Italy).

### Cyclic adenosine monophosphate (cAMP) assay

cAMP was measured using the ELISA-based cAMP XP assay kit (Cell Signaling Technologies, Euroclone, Milan, Italy) according to the manufacturer’s protocol. Briefly, WT and GPER KO MDA-MB-231 cells (100 × 10^3^ cells/well) were seeded in 96 well plate and then switched to 1% charcoal-stripped FBS for 36 h, treated with 10 µM Forskolin for 30 min where required, and then lysed in 100 μl of kit cell lysis buffer. After centrifugation (20,000 × *g* × 5 min at 4 °C), 50 μl aliquots of supernatant were taken and added to 50 μl of horseradish peroxidase-cAMP kit solution for cAMP measurement. Quantification was based on a linear concentration curve, established with external cAMP standards. The plates were read at 450 nm on a Microplate Spectrophotometer Epoch™ (BioTek, AHSI, Milan Italy).

### Proliferation and anchorage-independent soft-agar colony formation assay

WT and GPER KO MDA-MB-231 (1 × 10^4^) were seeded in 24-well plates in regular growth medium, washed once they had attached, incubated in medium containing 2.5% charcoal-stripped FBS, the medium was renewed every 2 days. The proliferation rate was calculated counting the cells on day 5 using the Countess Automated Cell Counter, as recommended by the manufacturer’s protocol (Thermo Fisher Scientific, Milan, Italy).

To evaluate the soft-agar colony-forming ability of WT and GPER KO MDA-MB-231 cells, DMEM/F12 (Life Technologies, Milan, Italy), a layer of 0.6% noble agar (Merck Life Science, Milan, Italy) in DMEM/F12 supplemented with 5% FBS was first laid in tissue culture plates and allowed to solidify at room temperature as the “base agar”. Subsequently, the cells were mixed with 0.3% noble agar in DMEM/F12 supplemented with 5% FBS as the “cell agar” laid on top of the “base agar” layer. Once the “cell agar” solidified, a layer of 0.3% noble agar in DMEM supplemented with 5% FBS was added as “feeder agar”. The medium on top was refreshed every week of culturing. The colonies were visualized after about 2 weeks of culturing, by normal light microscopy, and the resultant images were analyzed using the WCIF ImageJ software.

### Cell cycle analysis

To determine cell cycle distribution analysis, WT and GPER KO MDA-MB-231 cells (1 × 10^5^) were cultured in regular medium in six- well plates and shifted in 1% charcoal-stripped FBS for 36 h. Next, the cells were pelleted, once washed with PBS and fixed in 50% methanol overnight at −20 °C and stained with a solution containing 50 μg/ml propidium iodide (PI), 20 U/ml RNAse-A and 0.1% Triton (Merck Life Science, Milan, Italy). Cell phases were estimated as a percentage of a total of 10,000 events. Samples were then analyzed with CytoFLEX flow cytometry (Beckman-Coulter, Milan, Italy).

### Colony formation assay

WT and GPER KO MDA-MB-231 cells were cultured in regular growth medium to 90% confluence. Cells were then trypsinized, counted, and seeded (1 × 10^3^) in six-well plates in medium containing 2.5% charcoal-stripped FBS. The medium was renewed every 3 days. After 10 days, cells were washed with PBS, fixed in acetone:methanol (1:1) for 3 min at room temperature and then stained with Giemsa for 10 min. A total of 10 pictures for each condition was detected using a digital camera and colony number was measured by ImageJ software.

### Mammosphere formation assay

Phenol red-free DMEM/F12 (Merck Life Science, Milan, Italy) containing B27 supplement (Gibco, Life Technologies, Milan, Italy) and EGF (20 ng/ml), was used as mammosphere medium to culture WT and GPER KO MDA-MB-231 cells in a low-attachment 6 plate. Specifically, cells (500 cells/cm2) were plated as a single cell suspension using a 25 G needle and 2 ml of mammosphere medium was added to each well, the cells were incubated in a humidified atmosphere at 37 °C and 5% CO_2_ for 5 days without moving or disturbing the plates and without replenishing the medium. After 5 days, the number of mammospheres greater than 50 μm diameter was counted (at ×40 magnification). For secondary generation of mammospheres, the medium containing the mammospheres (at least 100 mammospheres) was collected and after a wash with PBS and a centrifuge at 115 × *g* for 5 min, the pellet was resuspended in 300 μl of 0.5% trypsin/0.2% EDTA and disaggregated using a 25 G needle to produce single cell suspension. Next, the cells were centrifuged, the pellet was resuspended in 2 ml of mammosphere medium per well and seeded in low-attachment 6 plates at the same seeding density that was used in the primary generation. The cells were incubated in a humidified atmosphere at 37 °C and 5% CO_2_ for the same number of days as the primary culture (3–12 days) without replenishing the media. Mammosphere forming efficiency (%) was calculated as follows: (number of mammospheres per well/number of cells seeded per well) × 100.

### Migration assays

Transwell 8 μm polycarbonate membranes (Costar, Merck Life Science, Milan, Italy) were used to evaluate in vitro cell migration. CAFs (5 × 10^4^) were seeded in the upper chamber in 300 μL serum-free medium. Conditioned medium of WT and GPER KO MDA-MB-231 cells was added to the bottom chambers in the presence or absence of 100 nM CTGF, where required. 12 h after seeding, cells on the upper surface of the membrane were removed by wiping with Q-tip, and migrated cells were fixed with 100% methanol, stained with Giemsa (Merck Life Science, Milan, Italy), photographed using a digital camera and counted using the WCIF ImageJ software.

### In vivo studies

Female 45-day-old athymic nude mice (nu/nu Swiss; Envigo Laboratories) were maintained in a sterile environment. At day 0, 14 mice were divided into two groups: the first (*n* = 7) were implanted in mammary fat pad in 0.1 mL of Matrigel (Cultrex, Trevigen Inc.) with exponentially growing of WT MDA-MB-231 cells (2 × 10^6^ per mouse), the second (*n* = 7) with GPER KO MDA-MB-231 cells. Mice well tolerated all in vivo procedures, since no changes in body weight, motor function or food and water consumption were observed. WT and GPER KO MDA-MB231 xenograft tumor growth was measured twice a week by caliper, along two orthogonal axes: length (L) and width (W). Tumor volumes (in cubic centimeters) were estimated by the following formula: TV = L × (W2)/2. At 28 days of growing, the animals were killed following the standard protocols and tumors were dissected from the neighboring connective tissue. Half of each tumor was flash frozen in liquid nitrogen, and the other half was fixed in 10% formalin for 24 h prior to paraffin embedding for subsequent the histologic analyses.

### Histologic analysis and immunohistochemistry

Formalin-fixed, paraffin-embedded (FFPE) sections of tumor xenografts were cut at 5 μm, were mounted on slides precoated with poly-lysine, air dried, deparaffinized, rehydrated (7–8 serial sections) and stained with hematoxylin and counterstained with eosin Y (Bio-Optica, Milan, Italy). Immunohistochemical experiments were performed after heat-mediated antigen retrieval. Hydrogen peroxide (3% in distilled water) was used, for 30 min, to inhibit endogenous peroxidase activity while normal goat serum (10%) was utilized, for 30 min, to block the non-specific binding sites. The epithelial nature of the tumors was verified by immunostaining with anti-human cytokeratin 18 antibody (1:100; Santa Cruz Biotechnology, Milan, Italy). Tumor sections were immune labeled with Ki67 (1:100; DAKO, DBA, Milan, Italy), which served as a proliferation marker. All sections were incubated with primary antibodies at 4 °C overnight. Then, a universal biotinylated IgG was applied (1:500) for 1 h at room temperature, followed by ABC/HRP. Immunoreactivity was visualized using DAB. For negative controls, nonimmune serum is replaced at the same concentration of the primary antibody. Sections nuclei were also counterstained with hematoxylin. For each sample six-seven serial sections were processed and visualized using an OPTIKA XDS-3 microscope (OPTIKA Microscopes, Italy) and the images were acquired with OpticalSview software using an OPTIKA 4883.13 CAM.

### Statistical analysis

The statistical analysis was performed using ANOVA followed by Newman Keuls’ test to determine differences in means. Wilcoxon-Mann-Whitney test was used for the statistical comparisons for in vivo studies. *p* < 0.05 was considered statistically significant.

### Supplementary information

Supplementary Figure 1

Supplementary Table 1

Supplementary Table 2

Supplementary Material

## Data Availability

The data that support the findings of this study are available from the corresponding author upon reasonable request. Full length uncropped original western blots used in the manuscript are provided in Supplementary File 1.

## Code availability

Bioinformatics analyses were performed on R Studio (version 4.1.3). The full R code employed is available from the corresponding author upon reasonable request.
