# Supplementary material for: GPER deletion triggers inhibitory effects in triple negative breast cancer (TNBC) cells through the JNK/c-Jun/p53/Noxa transduction pathway
Source: Cell Death Discov. 2023 Sep 26;9:353. doi: 10.1038/s41420-023-01654-0 (PMC10520078; doi:10.1038/s41420-023-01654-0)
Supplement: Supplementary file 1 — Supplementary Figure 1 [file 41420_2023_1654_MOESM1_ESM.pdf]

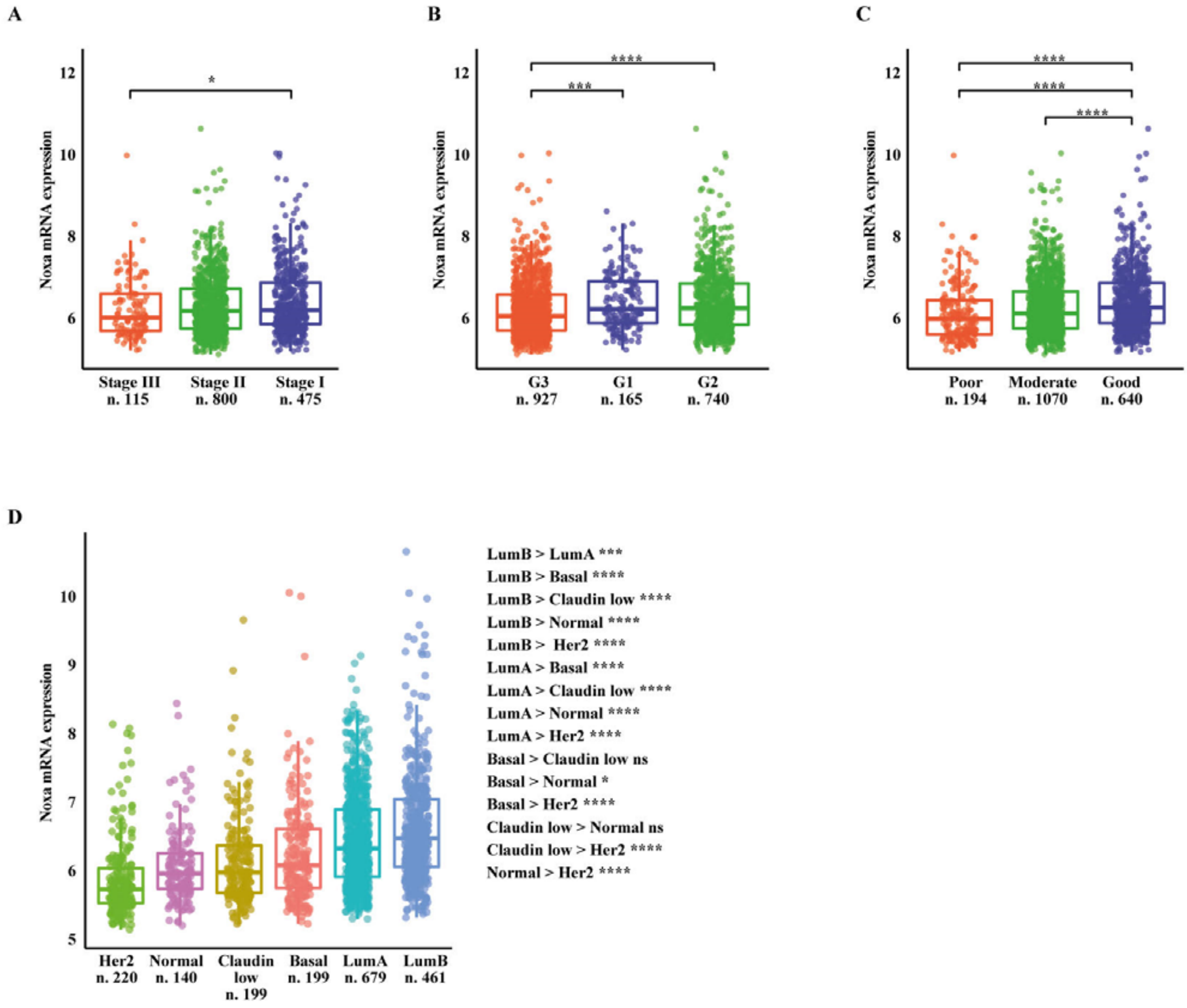

Supplementary Figure 1. Boxplots of Noxa mRNA levels in the METABRIC cohort of breast cancer patients stratified by tumor stage (A) and grade (B). (C) Boxplot showing the trend between the expression of Noxa and the determined Nottingham prognostic index of the METABRIC breast tumors. (D) Noxa mRNA levels according to the breast cancer intrinsic molecular subtypes of the METABRIC cohort. G3, Grade 3; G1, Grade 1; G2, Grade 2; Lum A, Luminal A; Lum B, Luminal B; ns, not significant; (*) indicates $p < 0.05$, (***) indicates $p < 0.001$ and (****) indicates $p < 0.0001$.
