# Supplementary material for: GPER deletion triggers inhibitory effects in triple negative breast cancer (TNBC) cells through the JNK/c-Jun/p53/Noxa transduction pathway
Source: Cell Death Discov. 2023 Sep 26;9:353. doi: 10.1038/s41420-023-01654-0 (PMC10520078; doi:10.1038/s41420-023-01654-0)
Supplement: Supplementary file 2 — Supplementary Table 1 [file 41420_2023_1654_MOESM2_ESM.pdf]

ID	Description	Enrichment Score	p-value
GO:0042127	regulation of cell population proliferation	-0.34833415	0.041958042
GO:0048699	generation of neurons	-0.359675021	0.035964036
GO:0032989	cellular component morphogenesis	-0.417821982	0.026973027
GO:0048666	neuron development	-0.382163291	0.033966034
GO:0098609	cell-cell adhesion	-0.475752729	0.000257468
GO:0008284	positive regulation of cell population proliferation	-0.410227248	0.010088439
GO:0032990	cell part morphogenesis	-0.410696831	0.044955045
GO:0048858	cell projection morphogenesis	-0.410696831	0.044955045
GO:0030155	regulation of cell adhesion	-0.392152365	0.030969031
GO:0120039	plasma membrane bounded cell projection morphogenesis	-0.406520347	0.048951049

Supplementary Table 1. List of the biological process (BP) terms from the gene ontology (GO) analysis of the down-regulated genes in GPER KO MDA-MB-231 cells respect to WT MDA-MB-231 cells.
