# Supplementary material for: GPER deletion triggers inhibitory effects in triple negative breast cancer (TNBC) cells through the JNK/c-Jun/p53/Noxa transduction pathway
Source: Cell Death Discov. 2023 Sep 26;9:353. doi: 10.1038/s41420-023-01654-0 (PMC10520078; doi:10.1038/s41420-023-01654-0)
Supplement: Supplementary file 3 — Supplementary Table 2 [file 41420_2023_1654_MOESM3_ESM.pdf]

ID	Description	Enrichment Score	p-value
GO:0010942	positive regulation of cell death	0.283649287	0.00234939
GO:0051276	chromosome organization	0.288271424	0.00539124
GO:1901615	organic hydroxy compound metabolic process	0.249854748	0.01382484
GO:0043065	positive regulation of apoptotic process	0.289432575	0.01008844
GO:0043068	positive regulation of programmed cell death	0.271889626	0.01218082
GO:0042592	homeostatic process	0.170467472	0.03396603
GO:0055085	transmembrane transport	0.201281191	0.02497503
GO:1905114	cell surface receptor signaling pathway involved in cell-cell signaling	0.232471464	0.03496504
GO:0010876	lipid localization	0.302787383	0.00784661
GO:0016055	Wnt signaling pathway	0.257682709	0.02497503

Supplementary Table 2. List of the biological process (BP) terms from the gene ontology (GO) analysis of the up-regulated genes in GPER KO MDA-MB-231 cells respect to WT MDA-MB-231 cells.
