# Supplementary material for: GPER deletion triggers inhibitory effects in triple negative breast cancer (TNBC) cells through the JNK/c-Jun/p53/Noxa transduction pathway
Source: Cell Death Discov. 2023 Sep 26;9:353. doi: 10.1038/s41420-023-01654-0 (PMC10520078; doi:10.1038/s41420-023-01654-0)

Figure 1 panel B

GAPDH GPER

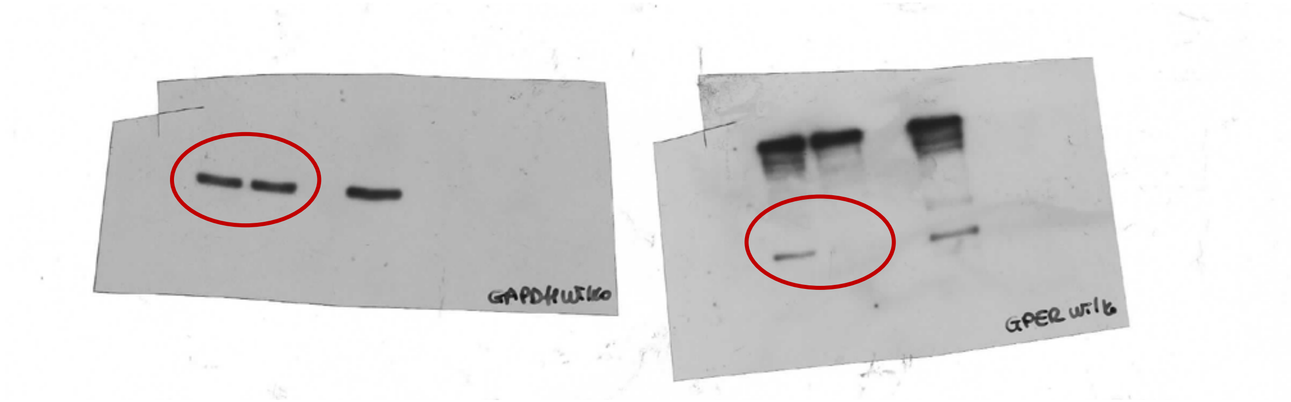

Figure 4 panel C

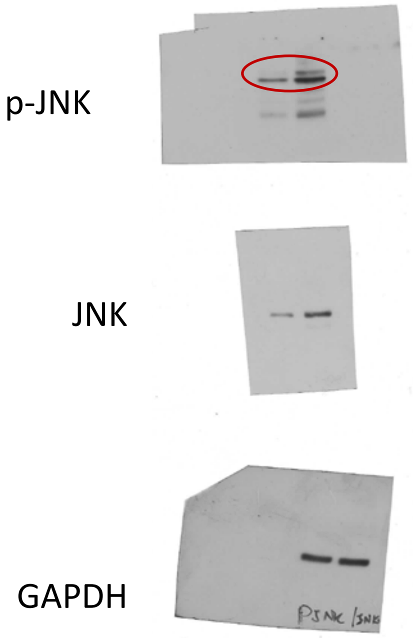

Figure 4 panel D

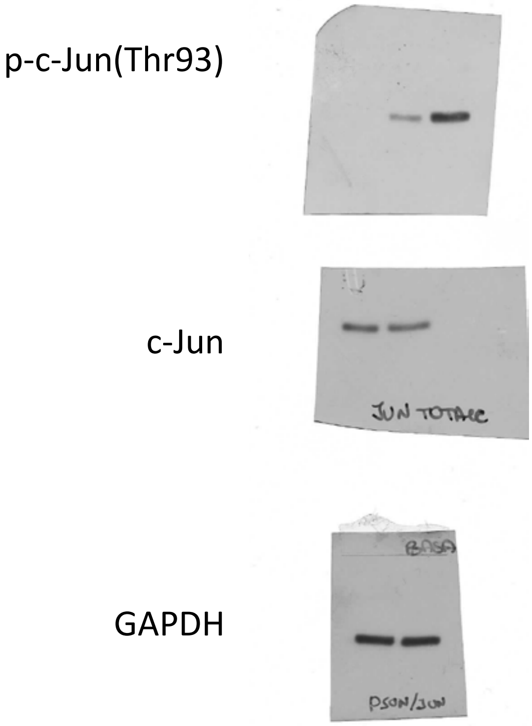

Figure 4 panel F

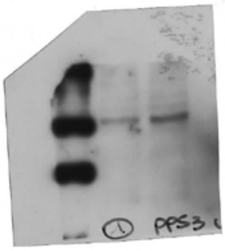

p-p53 (Ser15)

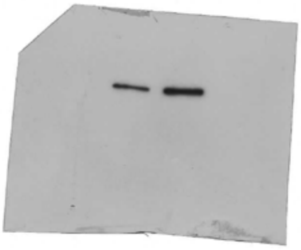

p53

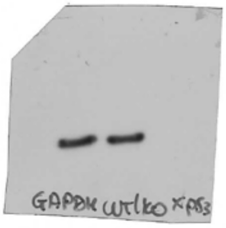

GAPDH

Figure 4 panel G

p-c-Jun (Thr93)

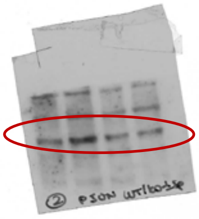

Jun

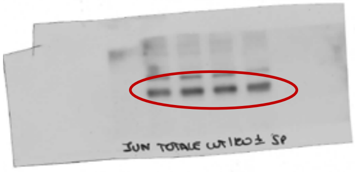

p-p53 (Ser15)

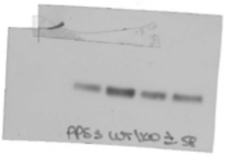

p53

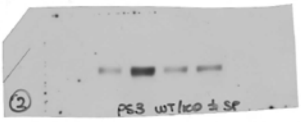

GAPDH

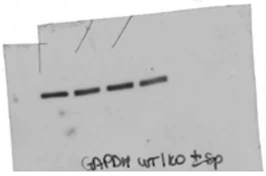

Figure 4 Panel I

p-JNK

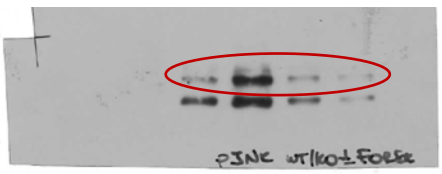

JNK

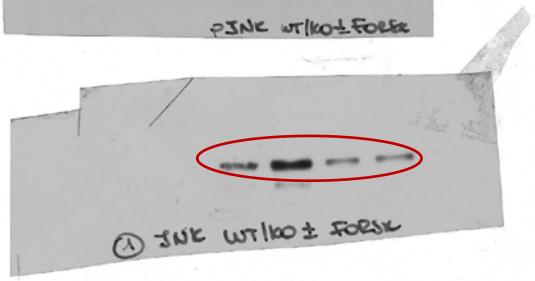

p-c-Jun (Thr93)

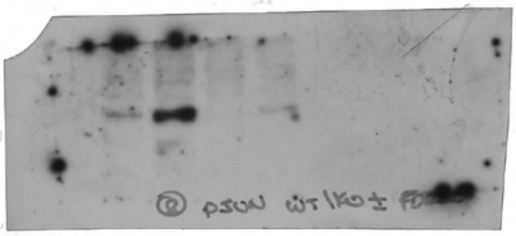

c-Jun

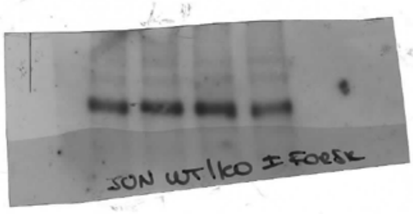

p-p53 (Ser15)

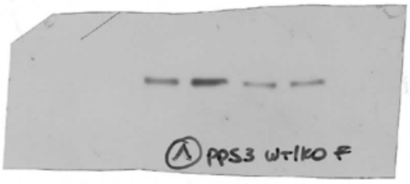

p53

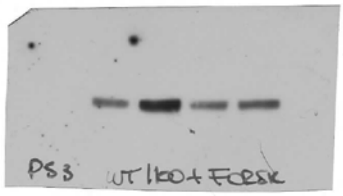

GAPDH

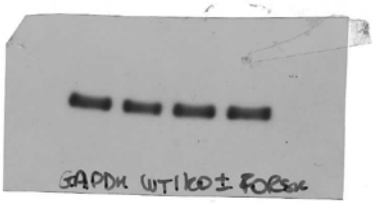

Figure 5 panel B

NOXA

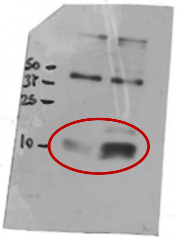

GAPDH

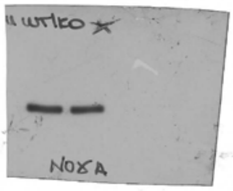

Figure 5 Panel C

NOXA

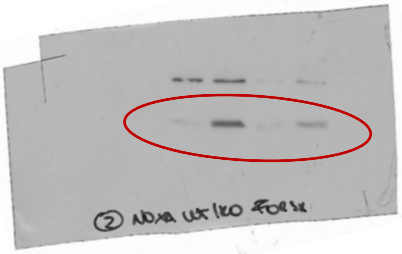

GAPDH

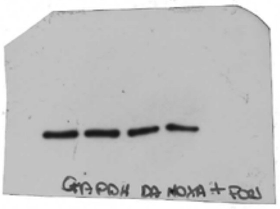

Figure 5 Panel D

NOXA

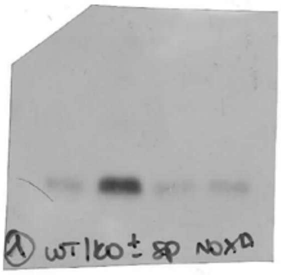

GAPDH

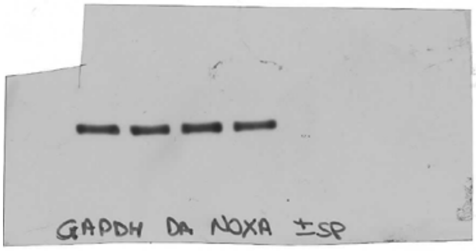

Figure 5 Panel E

NOXA

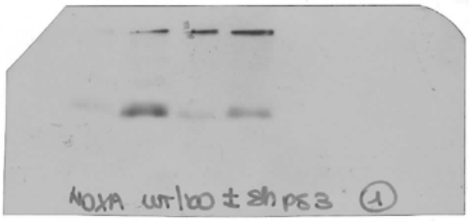

GAPDH

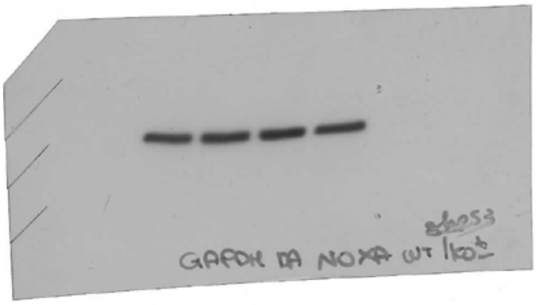

Figure 5 Panel F

P53

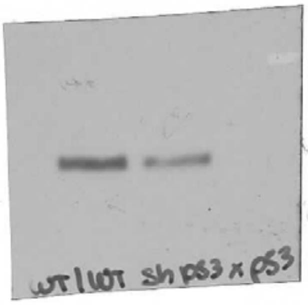

GAPDH

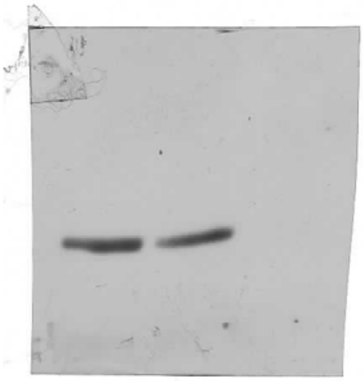

Figure 5 Panel H

NOXA

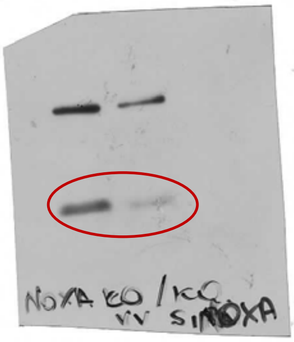

GAPDH

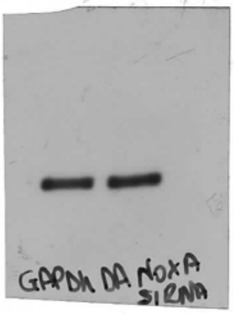

Figure 6 Panel B

CTGF

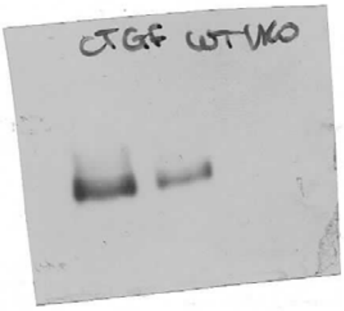

GAPDH

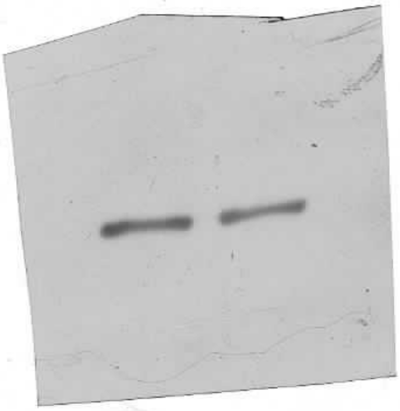

Figure 6 Panel C

CTGF

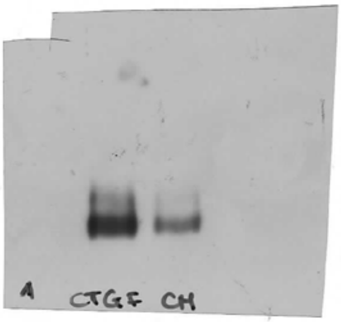

Supplement: Supplementary file 4 — Supplementary Material [file 41420_2023_1654_MOESM4_ESM.pdf]
